# Upcycling polyethylene terephthalate wastes for enhancing the performance of polyester from rice straw polyol in HDPE-composites

**DOI:** 10.1038/s41598-023-40031-w

**Published:** 2023-08-25

**Authors:** Emad S. Shafik, Vivian F. Lotfy, Altaf H. Basta

**Affiliations:** 1 Polymers & Pigments Dept., National Research Centre, Dokki, 12622 Giza Egypt; 2 Cellulose & Paper Dept., National Research Centre, El Buhouth St., Dokki, 12622 Giza Egypt

**Keywords:** Environmental sciences, Materials science

## Abstract

Upscaling the utilization of polymer wastes together with the valorization of undesirable waste rice straw (RS) will minimize the environmental impact of waste disposal by traditional tools. This present work assesses the utilization of polyethylene terephthalate wastes in enhancing the production of polyester-(high density polyethylene) HDPE from Rice straw polyol composites. In this respect, the polyester from rice straw polyol in hybrid with glycolysis polyethylene terephthalate wastes (Gly-WPET) was assessed in comparison with that resulted from RS-polyol, using FTIR, non-isothermal analysis, and mechanical tests. The data showed the positive role of Gly-WPET in hybrid with RS-polyol in production polyester with high thermal stability and mechanical properties. It provided an increase in activation energy of degradation, elongation, Young's Modulus, and modulus of toughness from 184.5 to 1201 kJ/mole, from 4.7 to 9.8%, from 47.5 to 66.5 MPa, and from ~ 4.0 to 23 J/m^3^, respectively. This behavior was reflected in the properties of HDPE-polyester polyol (PEPO) composites, especially in improving elongation (from 55.4 to 72%). These promising data persuade us to recommend the influential role of Gly-WPET in using PEPO from liquefied RS as a plasticizer.

## Introduction

Minimizing the solid wastes (e.g., plastics and agricultural) is essential for preserving the environment from traditional tools used to dispose of these wastes, which affects human health. Hence, the disposal of agricultural solid waste is one of the most pressing environmental issues^1^. Polyethylene terephthalate waste (WPET) is an example of the most important commercially consumed plastics in our daily life. On the other hand, rice straw (RS) is regarded as undesirable agricultural waste. These wastes are in massive amounts, and most are not fully utilized. Therefore, this waste management with the upcycling concept has become an important social issue. There are mainly two ways for PET recycling, which can be done mechanically or chemically. Mechanical recycling is easy to employ but presents some limitations as the properties of the final product decrease from the second cycle, while chemical recycling offers versatile procedures. The potential for chemical recycling is that it enables an integrated recycling process, where PET is depolymerized to its original constitution allowing the synthesis of new high value-added products^2,3^.

Upgrading the utilization of undesirable agro-fibers as precursors for the production of carbon materials (activated carbon and carbon nanotubes), functional paper, artificial wood, hydrogels for agricultural purposes, as well as nanoparticles for controlling the release of fertilizer as well as bioactive and optical compounds^4–12^, is a beneficial approach, also for the production of low-cost products. Agricultural wastes in polymer composites have received interest in academic and industrial sectors. Many types of natural fibers have been used to reinforce the polymer composites^13–16^. The thermoplastics polymers used as matrices in the production of composites with natural fibers are high density (HDPE) and low density (LDPE) polyethylene, chlorinated polyethylene (CPE), polypropylene (PP), normal polystyrene (PS), and polyvinylchloride (PVC). Unfortunately, the relatively poor compatibility between the composite components noticed in the scanning electron microscopy (SEM) study^17^ is a drawback. Different methods are performed to improve the compatibility by modifying the fiber matrix via adding coupling agents or accepting the fiber’s hydrophobic properties by grafting with hydrophobic monomers or esterification^15,16,18,19^. Moreover, extracting hemicellulose from fibers enhances its performance for improving the produced composite's water resistance and tensile strength of the produced composite^20^.

Polymer–polymer composites are also reported in literature. Blending polyolefins with polyethylene terephthalate (PET) has involved significant study activity. Guerrero et al.^21^ observed that the blending of PET increased the tensile strength property of HDPE; however, the reverse trend was noticed in the case of the elongation property. Studying the composite from recycled HDPE and PET in the presence and absence of compatibilizer, using a co-rotating twin screw extruder, proved to be effective in progressively increasing the interactions between two phases and enhancing the phase dispersion of the blends^22^. The mechanical properties of HDPE-based composites were also improved by adding sawdust, polyethylene-*g*-maleic anhydride, and commercial alumina additives. These additives also improved thermal and flammability properties^23^.

Continuing our work on the valorization of lignocellulosic-byproducts in the production of valuable products, such as, carbon nanostructures, functional wood and paper as well as hydrogels for agricultural purposes and controlled release systems^4–7,9,23^, the current work was focused on enhancing the RS-based polyol in preparation of polyester polyols (PEPO) and further applied in HDPE-composites. The synergistic effect of glycoside polyethylene terephthalate waste (Gly-WPET) on RS-Polyol was evidenced from increasing the thermal stability and mechanical properties (tensile strength, elongation, young's modulus, and toughness) of PEPO. Additionally, it promotes the application of PEPO as a plasticizer and in the production of high-performance HDPE-PEPO composites.

## Experimental

### Materials and raw materials

Rice straw with the following chemical constituents: 43.6% α-cellulose, 20.4% Lignin, 13.0% hemicellulose and 15.9% ash, was collected from the field-Lower Egypt. Ethylene glycol M.W 62.07 and Adipic acid M.W 146.14 were purchased from Alpha Chemika, India. Sulfuric acid M.W. 98.07 and sodium hydroxide were obtained from ADWIC co., 1,4-Dioxane MW 88.11 were purchased from s.d. fine-chem limited, Imidazole M.W 68.08 was obtained from Sisco Research Laboratories, India. Zinc acetate MW 183.48 days 1.84 g/mL was obtained from Aldrich. phthalic anhydride M.W 148.12 days 1.53 g/mL tin (II) ethyl hexanoate M.W 405.12 g/mol, and density 1.251 g/mL. 1,6-diisocyanatohexane was obtained from across company. Cobalt naphthenate commercial grade was purchased from Morgan chemical company (Abbasia, Egypt). High density polyethylene (HDPE) was delivered from Hebei Di warm technology company, China, with softening point of 125–135 °C. Silica filler was supplied by the Transport and Engineering Company, Alexandria, Egypt.

### Preparation of polyester polyols

#### Liquefaction of rice straw

Rice straw was passed through the following stages before being used: washed, milled by a hummer sieve 1.0 mm, and dried in an oven at 10 ± 2 °C for 24 h to remove moisture. Next, ethylene glycol (100 g) and sulfuric acid (3 g) were added into a three-neck flask equipped with a reflux condenser, a thermometer, and a stirrer. The flask was immersed in an oil bath preheated to 160 °C, and then the RS (30 g) was added with continuous stirring during the liquefaction process. After 4 h, the flask was immersed in cold water to quench the reaction, and the liquefied product was collected for use. The liquefied product was characterized by FTIR spectroscopy. The liquefaction yield was calculated according to the following equation, while the acid value and hydroxyl number for liquefied rice straw were evaluated according to Yao et al.'s method^24^.

$$Liquefaction \, yield, \%=\left[1- \frac{Dr \, mass \, of \, solid \, residue}{ Dry \, mass \, of \, feedstock}\right] \times 100$$

#### Glycolysis of waste poly (ethylene terephthalate) (Gly-WPET) drinking bottles

The WPET was obtained from waste soft drink bottles, followed by cutting them into pieces. It was then washed and dried. PET and EG (1:4) (wt/wt) were charged, and 0.5% zinc acetate was added as a catalyst. The glycolysis reaction was carried out at 180–200 °C under reflux in the nitrogen atmosphere for about 4 h. The reaction product was then quenched on ice. The solid precipitate was separated and washed with hot water. The product was later washed with ethyl acetate to remove unreacted PET. Finally, the purified product was dried and stored^25^.

#### Preparation of polyester polyols from hybrid RS-polyol and Gly-WPET

The RS-Polyol produced in sub-title 2.2.1 was used to synthesize different polyol-based polyester individually and in hybrid with Gly-WPET. The reagents used were Adipic acid, phthalic anhydride, and 0.5% of tin 2 ethyl hexanoate catalyst. as shown in Table 1. The reaction was carried out in a reactor equipped with a stirrer, thermometer, nitrogen inlet, and reflux set. This esterification reaction was carried out at 180–190 °C, and the polyester product was then cooled and stored.

**Table 1:** Formulations of polyester polyols.

Starting material charge in grams	Formulations samples					
	E0	E10	E20	E30	E50	E100
Adipic acid	21.2	21.2	21.2	21.2	21.2	21.2
Phthalic anhydride	3.1	3.1	3.1	3.1	3.1	3.1
Polyol rice straw	75.2	67.68	60.16	52.64	37.6	–
Gly-WPET	–	7.52	15.04	22.56	37.6	75.2
Tin 2-ethyl hexanoate	0.5	0.5	0.5	0.5	0.5	0.5
Total	100	100	100	100	100	100

#### Preparation of cured polyester polyols/silica composites

For preparing cured polyester polyol/silica composites, 1,6-diisocyanatohexane was added to polyester polyol with different ratios starting from 10% up to 50 wt%, together with a fixed ratio of silica filler (20%) and was added with a fixed ratio 20%. cobalt naphthenate (2%). The mixture was stirred well for 5 min to ensure a homogeneous distribution, then put into a mold with dimensions 10.3 cm × 0.8 cm and allowed to cure at room temperature.

### Characterization of polyester polyols

Different techniques were carried out to evidence the formation of polyesters and the accompanying difference from the use of glycolysis WPET-RS polyol hybrid.

- *FT-IR analysis* Infrared spectra were examined by using Fourier Transform Infrared Spectrophotometer (JASCO FTIR-6100E) (Japan). The samples were mixed with KBr and pressed as discs. The absorbance spectra were recorded in the wavenumber range 4000–400 cm^−1^.
- ^*1*^*H-NMR spectroscopy* ^1^H-NMR was measured in deuterated chloroform using the JEOL ECA 500 MHz NMR spectrometer.
- *Gel permeation chromatography (GPC)* The samples were measured using gel permeation chromatography (GPC) (PL-GPC-220, high temperature chromatography, Agilent Technologies, USA).
- *Thermogravimetric analysis (TGA)* was carried out for the pure and the loaded nanocomposite hydrogels using Setaram LABSYS EVO STA, France. All measurements were achieved over a temperature range of 30–600 °C using a heating scan rate of 10 °C/min in an inert atmosphere of nitrogen gas (30 mL/min). In addition, the kinetic parameters of thermal degradation were calculated according to References^26–28^.

### HDPE/PEPO composites preparation and testing

HDPE, polyester from RS-polyol, polyester from WPET, and their hybrid (50:50) were blended using weight ratios 100/0, 95/5, and 90/10 in an internal mixer (Brabender plasticorder) at 30 rpm and temperatures 170 °C. First, HDPE was added to the mixer, and the polyester was added after the polymers had reached their melt flow temperature, followed by silica in a fixed weight ratio (5%). The mixing process took 5 min on average. Next, the resultant composite was removed from the mixer, pressed into fixed dimensions (152 × 125 × 2.85 mm), using a laboratory hydraulic hot press at 170 °C for 5 min, and then cooled down to room temperature.

The mechanical measurements of polyesters and their composites, such as tensile strength, elongation at break, and modulus of toughness from stress–strain curves, were investigated using Zwick tensile testing machine (model Z010, Germany) according to ASTM D 638-03.

The morphology study was examined, after coating the samples with gold, by scanning electron microscopy using the Quanta instrument (model FEG250, FEI, Hillsboro, Oregon, USA), running the electron beam at 20 kV accelerating voltage.

## Results and discussion

### Characterization of RS-polyol and evidence of the polyesters formation

Liquefaction of rice straw with ethylene glycol using sulfuric acid as a catalyst resulted in polyol with an average yield of 58.7%, while the solid residue was 41.34%. This liquefaction yield approach was found by Liang et al.^29^ when they examined the liquefaction of crop residues in various solvents (ethylene carbonate, ethylene glycol, and polyethylene glycol). They found the liquefaction yield of crop residues using ethylene glycol was 60. Acid value and hydroxyl value were also estimated for liquefied rice straw, where acid value refers to the mass of potassium hydroxide (KOH) in mg required to neutralize one gram of examined compound. The acid and hydroxyl values for the prepared liquefied rice straw were 8.2 and 82.7 mg KOH/g, respectively.

**Figure 1:**
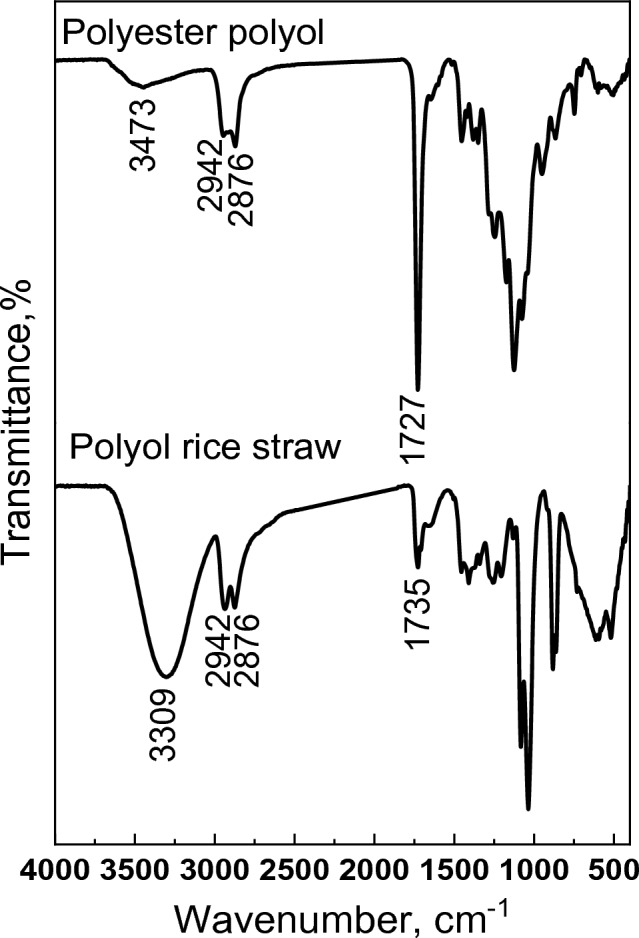
FT-IR of polyol rice straw and polyester polyol.

Different analyses were carried out for evidence of the formation of polyesters from RS-polyol and Gly-WPET, namely FTIR,^1^H NMR and molecular weight (MW) by GPC. The FT-IR spectra of RS-polyol (Fig. 1) showed a broad band at 3309 cm^−1^ indicating the presence of OH groups and the band at about 1050 cm^–1^ assigned to C–O–C asymmetry stretching. Observation of functional groups, such as –OH and C–O, implying that polyols were successfully prepared by liquefying rice straw under the studied conditions. The decrease in intensity of OH band on esterification with the sharp band at 1727 cm^−1^, which matched the carbonyl group, evidenced the modification of polyol to polyester. Concerning the spectra of Gly-WPET and its polyester.

**Figure 2:**
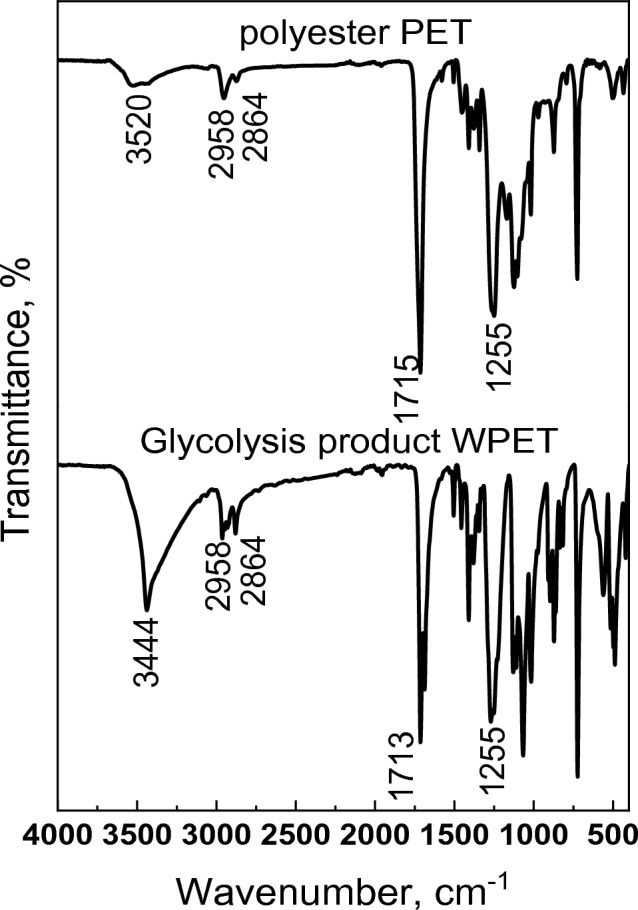
FT-IR of Gly-WPET and its polyester.

Figure 2 showed the following bands in the case of Gly-WPET: at 3437 cm^−1^ indicates the alcoholic group –OH; at 2985 cm^−1^ and 2864 cm^−1^ corresponds to the asymmetric and symmetric deformation of C–H stretching vibration, respectively. An intense band of the stretch carbonyl ester C=O is observed at 1713 cm^−1^, together with bands at 1267 cm^−1^ and 1099 cm^−1^ of asymmetric and symmetric vibration of C–O ester. Similar to the spectrum of polyester from RS-polyol the reduction in the intensity of OH groups (3520 cm^−1^) and increasing in the intensity of peak at 1713 cm^−1^ which related to carbonyl ester group was observed in spectrum of polyester from Gly-WPET.

**Figure 3:**
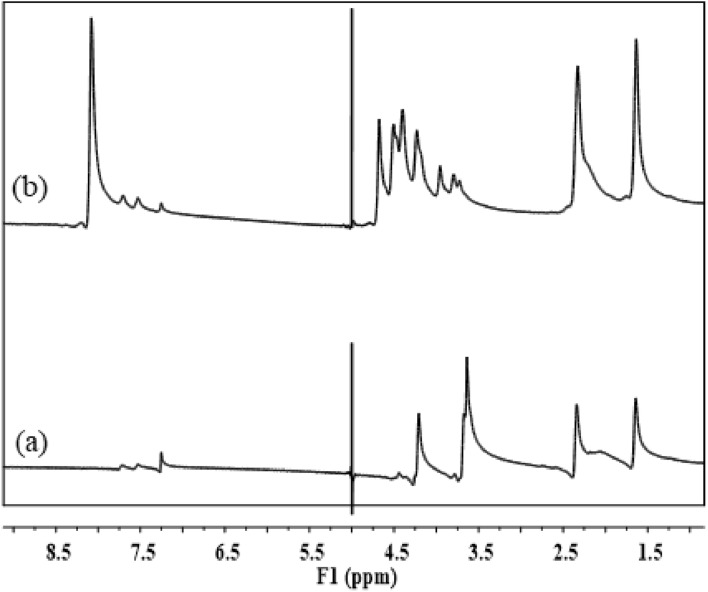
^1^H-NMR of prepared polyesters (**a**) from RS-polyol, and (**b**) from Gly-WPET.

The formation of polyesters from RS-polyol and Gly-WPET was also evidenced by ^1^H-NMR analysis. The chart of RS-polyol based polyester (Fig. 3a) showed the signal at 5.0 ppm for hydroxyl protons (CH_2_–OH), as well as signal at 3.6 ppm which is assigned to the methylene proton attached to the primary alcohol group (CH_2_–OH). The signal at 4.085 ppm referred to the CH_2_–O (C=O) proton, while the signal at 2.315 ppm referred to CH_2_–O (C=O). Due to protons CH_2_–CH_2_–O–(C=O), the signal at 1.62 ppm appears^30^. In the case of ^1^H NMR of polyester from the glycolysis product of WPET, Fig. 3b showed a signal due to the proton of aromatic CH_2_ of terephthalic acid at 8 ppm. The signal at 5.0 ppm is assigned to the hydroxyl protons (CH_2_-OH). Also, signals between 3.7 and 3.9 ppm are assigned to the methylene proton attached to the primary alcohol group, CH_2_–OH, while signals between 4.2 and 4.7 ppm and at 2.3 ppm are referred to the CH_2_–O (C=O), and CH_2_–(C=O) protons. respectively. Finally, the signal due to protons CH_2_–CH_2_–O–(C=O) appears at 1.64 ppm^16^.

The estimated number-average molecular weight (Mn), weight-average molecular weight (Mw), and polydispersity (PD = Mw/Mn) of polyesters from RS-polyol and its hybrid with glycolysis product of WPET (Gly-WPET) were recorded in Table 2. The total Mn and Mw ranged from 2.47 × 10^5^ to 3.14 × 10^5^ and from 2.74 × 10^5^ to 3.87 × 10^5^, respectively, during the esterification of liquefied rice straw and its hybrid with glycolysis product of WPET. Interestingly, using liquefied RS-WPET improved the monodisperse of the resulting polyester, where the PD (M_w_/M_n_) was decreased from 1.232 to 1.089–1.109.

**Table 2:** Molecular weight of polyesters from RS-polyol and its hybrid with glycolyzed WPET.

PEPO sample	GPC parameter		
	M_n_	M_w_	PD
PEPO from RS-polyol	3.14 × 10^5^	3.87 × 10^5^	1.232
PEPO from 90% RS-polyol + 10% Gly. WPET)	4.06 × 10^5^	4.42 × 10^5^	1.089
PEPO from 50% RS-polyol + 50% glycolysis WPET	2.47 × 10^5^	2.74 × 10^5^	1.109

### Optimizing RS-polyol/Gl-WPET hybrid-based polyesters

In this study, two effects were carried out to optimize the role of glycolysis WPET for preparing PEPO from RS-polyol, via changing the amount of 1,6-diisocyanatohexane as curing agents and the substitution ratio of polyol by glycolysis of WPET.

#### Effect of curing agent

**Figure 4:**
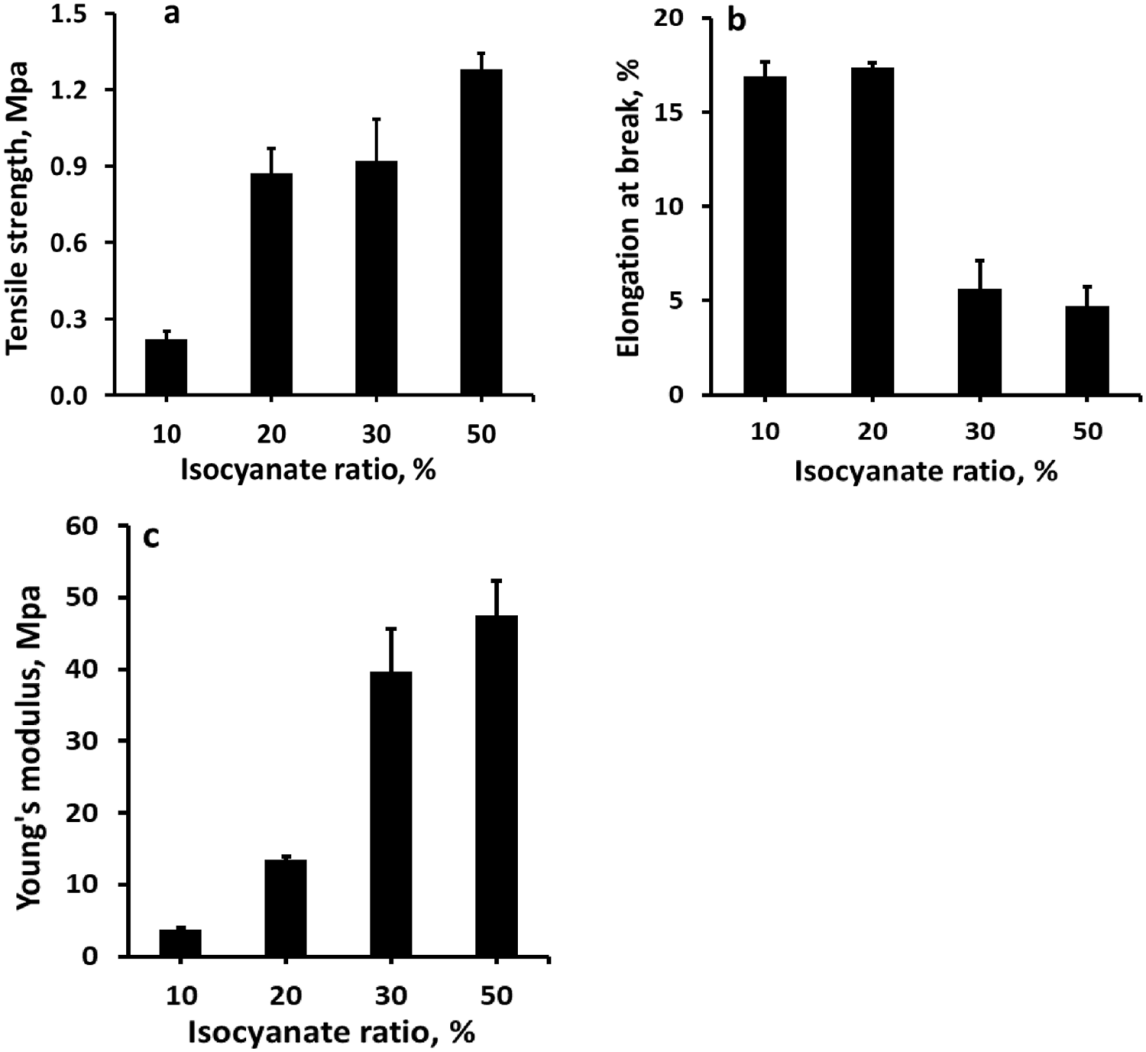
Mechanical properties of polyester polyol from rice straw versus amount isocyanate curing agent (**a**) Tensile strength, (**b**) elongation at break and (**c**) young’s modulus.

In this study different ratios of1,6-diisocyanatohexane (10, 20, 30 and 50%) were added to cure the polyester from RS-polyol to specify the optimum ratio, which performed polyester with high mechanical properties. Figure 4a–c illustrates the changes in mechanical properties, including tensile strength, elongation at break, young’s modulus, and modulus of toughness for prepared polyester versus the isocyanate ratio. The tensile strength increased gradually with increasing the isocyanate ratio from 10 to 50%, where the tensile strength and young's modulus increased from 0.22 to 1.28 MPa and from 3.77 to 47.47 MPa, respectively.

In contrast, the elongation at break was decreased with increasing the isocyanate ratio. The improvement in tensile strength and young's modulus may be ascribed to increased crosslinking density with increasing isocyanate ratios. The positive effect of curing agents on mechanical properties agrees with that reported by Lee et al.^25^.

#### Effect of substituting RS-polyol by Gly-WPET

**Figure 5:**
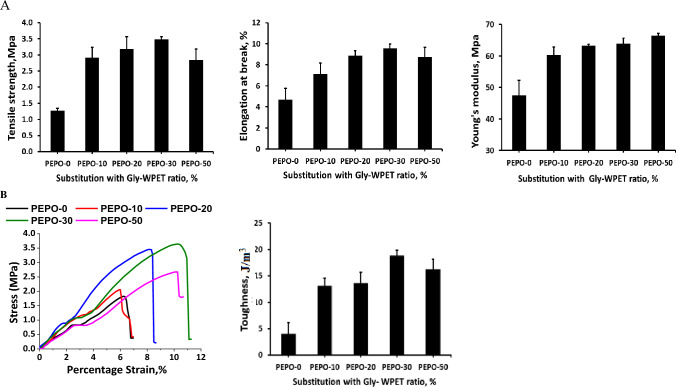
(**A**) Mechanical properties of polyester polyols from substituting the RS-polyol by Gly-WPET (**B**) toughness for stress–strain curve.

From the preceding data, 50% curing agent was provided from polyol with relatively high strength properties. This percentage was used for further study to evaluate the role of substituting RS-polyol by Gly-WPET on the properties of produced polyester. The obtained data are illustrated in Fig. 5, which shows the positive effect of replacing a part of RS-polyol by Gly-WPET. The tensile strength of polyester increased gradually with the substitution ratio of glycolysis WPET (increased from 1.28 MPa to 2.92, 3.19, and 3.7 for substitution 10%, 20%, and 30%, respectively. Further increase in the substitution ratio to 50% also improved the tensile strength of polyester (2.45 MPa) but less than 30% (Fig. 5a).

The elongation at break and Young's Modulus (Fig. 5a) increased with increasing the Gly-WPET till 50% (from 4.7 to 9.8% and from 47.47 to 66.48 MPa, respectively). As can be seen that the glycolysis of WPET provided improvement in modulus of toughness which was estimated from area of stress–strain curves (Fig. 5b), and the maximum improvement at 30% substitution [23.03 (J/m^3^)/control 3.98 J/m^3^].

**Figure 6:**
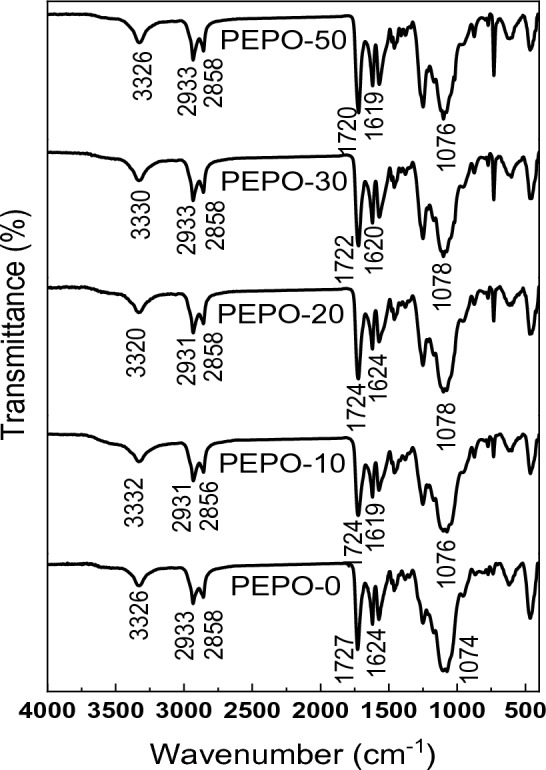
FTIR of polyester from RS-polyol and its hybrid with 10% (PEPO 10), 20% (PEPO 20), 30% (PEPO 30) and 50% (PEPO 50) Gly-WPET.

The influence of Gly-WPET on polyester from RS-polyol was also clear from FTIR spectra and TGA analysis. Figure 6 showed that the FT-IR spectra of all polyesters of hybrid liquefied samples included the same bands, but the only difference in increasing the intensity of bands at 1249 and 750 cm^−1^ with increasing the substitution ratio from 10 to 50%, which indicated the formation of a polymer network between polyester and curing agent and creation of the ether linkage. Moreover, the terephthalate group (OOCC_6_H_4_COO) was included in the product. The disappearance of peak N=C=O at 2250 cm^−1^ confirming that diisocyanate of curing agents completely reacted with polyester polyol^24^.

**Figure 7:**
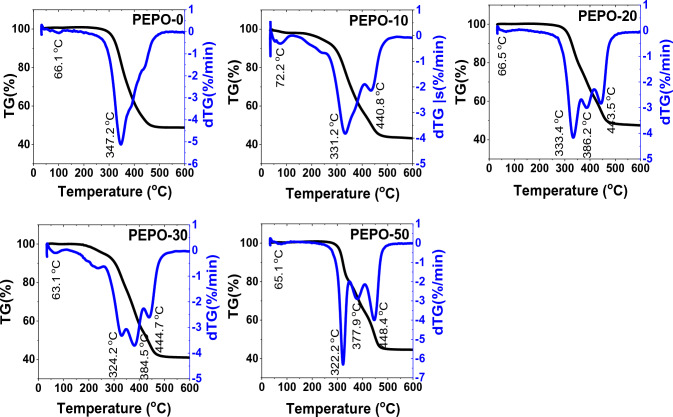
TGA and DTG curves of polyesters from RS-polyol and its hybrid with Gly-WPET.

Regarding the effect of substituting RS-polyol by Gly-WPET on the resulting polyester's thermal stability, Fig. 7 and Table 3 illustrated that the PEPO from liquefied RS was decomposed in two stages. First at lower than 100 °C, which was attributed to water dehydration, while the second at 250–500 °C regarded as the main degradation stage and related to the degradation of urethane linkage and bonds of network formation between polyester and the curing agent. The peak maximum of this main degradation peak appeared at 347.2 °C with an onset temperature of 301.0 °C. The resulting polyester from substituting the RS-polyol with Gly-WPET the decomposition proceeded in two and three stages. These additional stages are attributed to the dissociation of the interaction between RS-polyol, Gly-WPET, and curing agent with the formation of different strength bonds, in addition to the decomposition of the polymer chain of WPET, especially at a relatively high substitution ratio, which usually started to degrade at 375–400 °C according to the literature^31,32^. As can be noticed (Table 3), the percentage of residual weight increased with the substitution of PET from 37.7 to 45.5%. This is back to the fact that thermal pyrolysis of PET left much solid residue that did not decompose at examined temperature (600 °C). This is attributed to the interlinking reaction between the decomposed products of PET and with the formation of stabilized products^33^.

**Table 3:** TGA and DTGA measurements of the main degradation stage of polyesters from RS-polyol and its hybrid with different ratios od Gly-WPET.

Sample code	Decomposition step	Temp. range (°C)	DTG peak (°C)	Onset temp (°C)	Weight loss%	Ash % at 1000 °C	n order	R^2^	S_e_	E_a_ kJ/mol
PEPO-0	2nd	271.5–505.9	347.2	301.0	50.990	37.77	2.000	0.966	0.287	184.516
										∑ E_a_ = 184.5
PEPO -10	2nd	264.9–408.9	331.2	282.4	37.110	37.03	2.000	0.965	0.206	181.604
	3rd	405.4–483.9	440.8		12.360		2.000	0.953	0.219	424.532
										∑ E_a_ = 606.1
PEPO -20	2nd	271.3–364.7	333.4	293.0	22.617	45.56	2.000	0.965	0.227	273.677
	3rd	365.7–415.7	386.2		14.308		2.000	0.936	0.212	464.153
	4th	416.7–498.2	443.5		13.880		2.000	0.964	0.209	458.512
										∑E_a_ = 1196.3
PEPO -30	2nd	272.1–351.5	324.2	295.6	18.743	38.81	2.000	0.949	0.216	265.340
	3rd	354.4–415.5	384.5		20.445		2.000	0.941	0.235	394.969
	4th	413.7–480.5	444.7		13.037		2.000	0.953	0.220	500.428
										∑E_a_ = 1160.7
PEPO -50	2nd	289.9–347.5	322.3	303.9	19.704	41.47	1.500	0.993	0.064	301.328
	3rd	347.5–409.0	377.9		16.021		2.000	0.937	0.221	381.067
	4th	409.0–480.1	448.4		18.839		2.000	0.954	0.228	518.728
										∑E_a_ = 1201.1

The kinetic parameters (temperature range, DTG peak temperature, weight loss, correlation coefficient (R^2^), order "n", standard error estimate (S_e_), and activation energies (E_a_) of the main degradation in all samples are gathered in Table 3. In all cases, substituting RS-polyol with liquefied WPET provided lower onset temperature and DTG peaks than the stage of polyester from pure RS-polyol. At the same time, their activation energies increased gradually with increasing the substitution percentage. The activation energies of PEs from hybrid liquefied samples ranged from 606 to 1201 kJ/mol, while E_a_ of polyerster from pure RS-polyol was 184.5 kJ/mol). This indicated that liquefied WPET enhanced the bond formation during the esterification of RS-polyol, which provided thermal stabilization in the final product, which requires relatively higher E_a_ to degrade.

### Assessment of HDPE-PEPO composites

Blending polyolefins and polyesters have attracted considerable research because they are among the most consumed plastics. Therefore, HDPE was blended with the prepared polyesters. Blending these two materials could offer a very attractive balance of mechanical properties.

**Figure 8:**
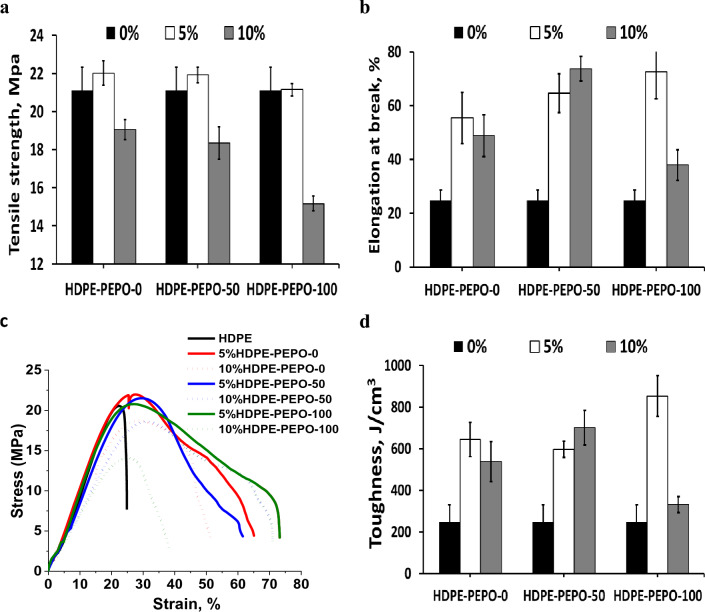
Mechanical properties of high-density polyethylene-polyester polyol (HDPE-PEPO) composites. (**a**) Tensile strength, (**b**) elongation, (**c**) stress–strain curve and (**d**) Toughness.

Figure 8 showed that incorporating the polyesters, especially 5%, from RS polyol and Gly-WPET, individually or in hybrid (50:50), positively affected the mechanical properties of HDPE. The improvement in elongation was more significant than the tensile strength, whereas the tensile strength improved from 21.1 MPa to 22.0, and 21.9 MPa, respectively. Above this ratio (10%), the tensile strength decreased. At the same time, the elongation at break increased from 24.8% for pure HDPE to be 55.4%, 64.6 and 72.0% for HDPE/5% polyester of RS-polyol, HDPE/5% hybrid polyesters, and HDPE/5% polyester WPET. As can be noticed, blending HDPE with 10% PEPO from hybrid RS-polyol with Gly-WPET, also improved the elongation as in the case of 5% WPET-PEPO. This promising behavior of 10% PE rather than 5% was also noticed in the case of toughness, where the area of stress–strain curve increased from 246.4 to 597.1 and 700.6 T (this is the wrong unit, should be J/cm^3^, please double check) for 5% and 10% PE blends. Based on the definition of plasticizer and the obtained data, we recommend the utilization of these PEPOs as a plasticizer.

**Figure 9:**
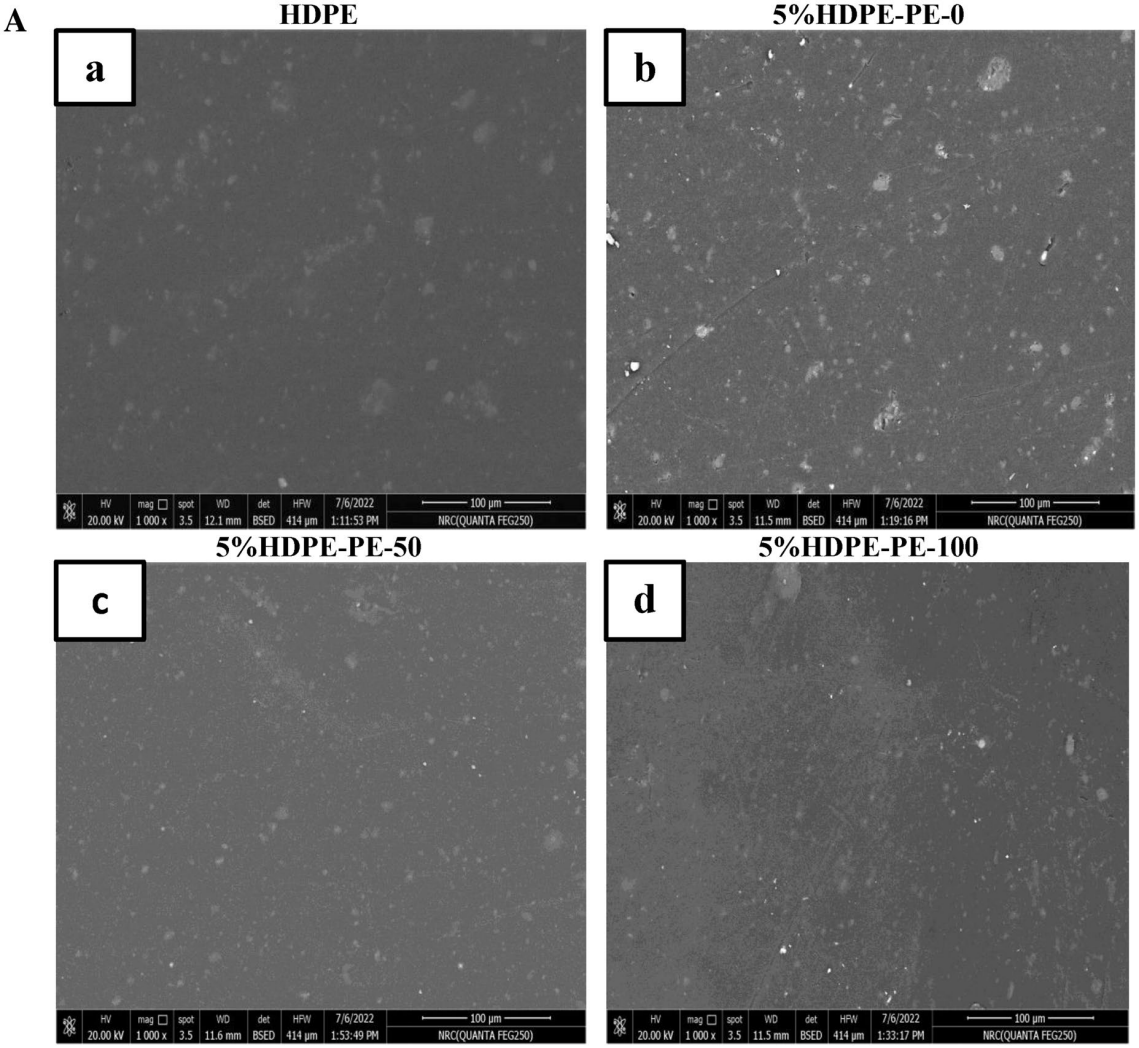

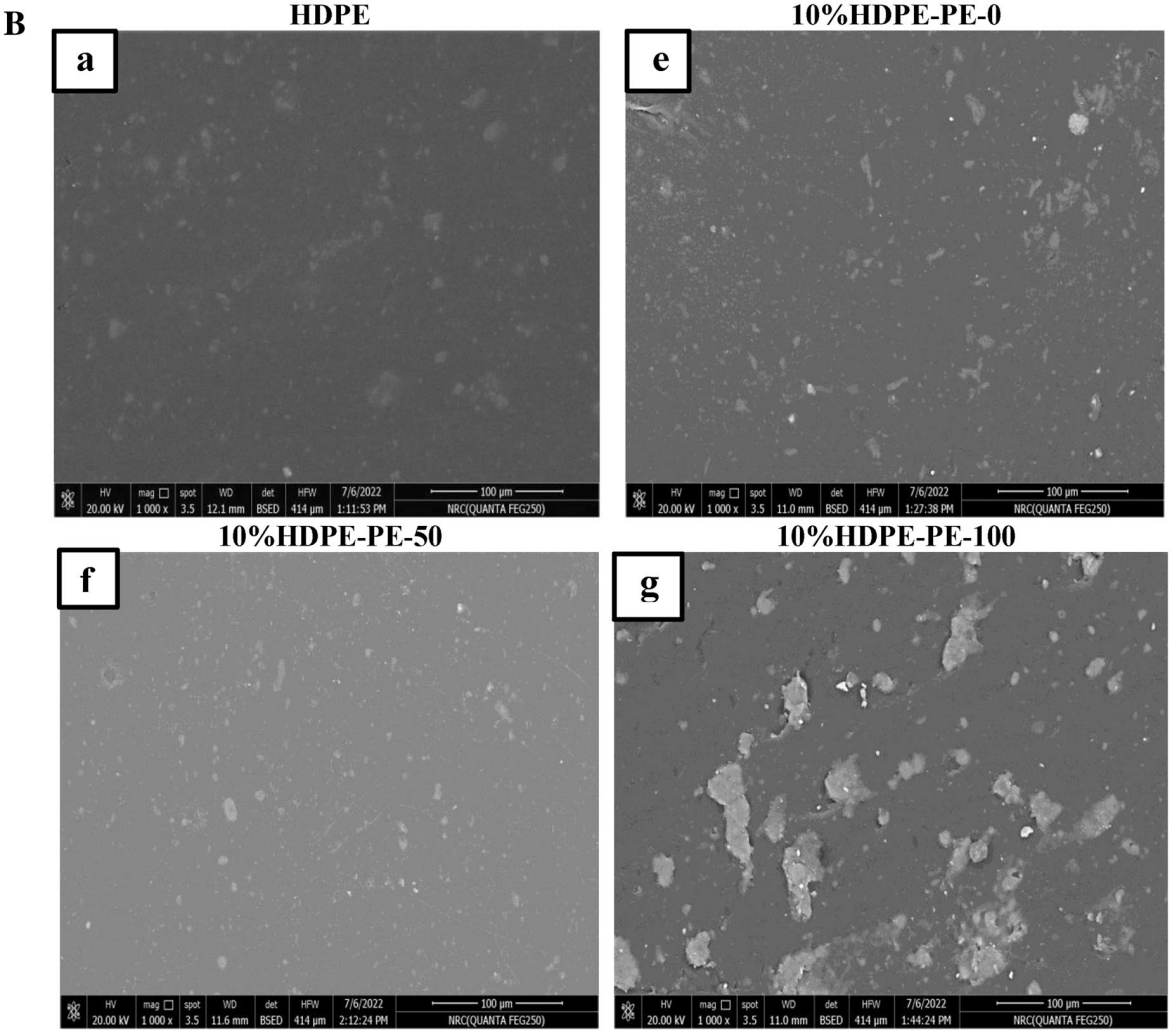
(**A**) SEM morphology of high-density polyethylene (HDPE) and HDPE blended with different types and 5% polyesters. (**B**) SEM morphology of high-density polyethylene (HDPE) and HDPE blended with different types and 10% polyesters.

The morphology of HDPE and HDPE-PEPO blends with different types and content from prepared polyesters is shown in Fig. 9A,B. Figure 9a clarifies a good dispersion of silica fillers on the HDPE matrix surface. Figure 9b–d illustrates the micrographs of HDPE–PEPO blends incorporating 5% PEPO from RS-polyol and Gly-WPET individually or in hybrid. These micrographs clarify the adhesion of polyester and silica fillers to the HDPE matrix was good at low content. The good adhesion results in enhancing the mechanical properties, which agrees with the other conclusion from our experimental results. In comparison, Fig. 9e–g showed that the adhesion of PEPO to the HDPE matrix was poor at high loading (10%). The weak adhesion results in damage or alteration of the mechanical properties. The SEM micrograph of higher PEPO loading showed some aggregates indicating inadequate additive dispersion within the HDPE matrix. These aggregates have become more present and more significant in size by increasing the ratio of polyester to 10%, especially in the case of WPET-100. This observation emphasized te trend of mechanical properties illustrated in Fig. 8.

## Conclusions

This work deals with promoting the utilization of bottle plastic wastes and undesirable rice straw in production of green products and avoiding the environmental risk from their disposing by burning. A potential route for using WPET to enhance the performance of polyester resulting from RS-polyol was evaluated. The production variables (amount of curing agent and replacing a percentage of RS-polyol by Gly-WPET) were optimized by estimating the thermal stability and mechanical properties of ester products. It was found that polyester from hybrid liquefied RS and Gly-WPET resulted in increase the thermal stability (E_a_ increased from 184.5 to 1201 kJ/mol), elongation (from 4.7 to 9.8%), young’s modulus (from 47.5 to 66.5 MPa), and toughness (~ 4.0 to 23 J/m^3^) than PEPO produced from RS-polyol. Additionally, it promotes the application of the PEPO as a plasticizer and in producing high-performance HDPE-polyester composites.

## Data Availability

All data generated or analyzed during this study are included in this published article.
